# 18β-Glycyrrhetinic acid induces human HaCaT keratinocytes apoptosis through ROS-mediated PI3K-Akt signaling pathway and ameliorates IMQ-induced psoriasis-like skin lesions in mice

**DOI:** 10.1186/s40360-020-00419-0

**Published:** 2020-06-03

**Authors:** Jintao Gao, Junfan Guo, Yuejuan Nong, Wenfei Mo, Huanan Fang, Jing Mi, Qi Qi, Mengjuan Yang

**Affiliations:** College of Biotechnology, Guilin Medical University, Guilin, 541100 Guangxi People’s Republic of China

**Keywords:** 18β-Glycyrrhetinic acid, Apoptosis, ROS, PI3K-Akt signaling pathway, Psoriasis

## Abstract

**Background:**

Psoriasis is a chronic inflammatory skin disease affecting 2–3% of the population worldwide. Hyperproliferative keratinocytes were thought to be an amplifier of inflammatory response, thereby sustaining persistence of psoriasis lesions. Agents with the ability to inhibit keratinocyte proliferation or induce apoptosis are potentially useful for psoriasis treatment. 18β-Glycyrrhetinic acid (GA), an active metabolite of glycyrrhizin, exhibits diverse pharmacological activities, including anti-inflammatory, anti-bacteria and anti-proliferation. The current study aims to evaluate the effects of GA on the proliferation and apoptosis of human HaCaT keratinocytes in vitro and investigate the effects of GA on the skin lesions of imiquimod (IMQ)-induced psoriasis-like mouse model in vivo.

**Methods:**

Cell viability was assayed by CCK-8. Flow cytometry was performed to measure apoptosis and reactive oxygen species (ROS), with Annexin V-FITC/PI detection kit and DCFH-DA probe respectively. Caspase 9/3 activities were measured using caspase activity assay kits. The protein levels of Akt and p-Akt were determined using Western blotting. IMQ was applied to induce psoriasis-like skin lesions in mice. The histological change in mouse skin lesions was detected using hematoxylin and eosin (H&E) staining. The severity of skin lesions was scored based on Psoriasis Area Severity Index (PASI). RT-PCR was employed to examine the relative expression of TNF-α, IL-22 and IL-17A in mouse skin lesions.

**Results:**

GA decreased HaCaT keratinocytes viability and induced cell apoptosis in a dose-dependent manner. In the presence of GA, intracellular ROS levels were significantly elevated. NAC, a ROS inhibitor, attenuated GA-mediated HaCaT keratinocytes growth inhibition and apoptosis. In addition, GA treatment remarkably decreased p-Akt protein level, which could be restored partially when cells were co-treated with GA and NAC. LY294002 (a PI3K inhibitor) treatment significantly enhanced GA-mediated cytotoxicity. Moreover, GA ameliorated IMQ-induced psoriasis-like skin lesions in mice.

**Conclusions:**

GA inhibits proliferation and induces apoptosis in HaCaT keratinocytes through ROS-mediated inhibition of PI3K-Akt signaling pathway, and ameliorates IMQ-induced psoriasis-like skin lesions in mice.

## Background

Psoriasis is a chronic immune-mediated skin disease resulting from genetic, epigenetic and environmental factors, which is characterized by hyperproliferation and aberrant differentiation of keratinocytes, excessive infiltration of leukocytes into the dermis or epidermis. It affects 2–3% of the population worldwide [1]. The exact pathogenesis of psoriasis still remains unclear, but dysregulated crosstalk between keratinocytes and immune cells is thought to contribute to psoriasis pathogenesis [2–4]. Several exogenous and endogenous triggers activate immune cells to release inflammatory cytokines, which is responsible for the typical unbalance between proliferation and differentiation processes in keratinocytes. Hyperproliferative keratinocytes respond to the cytokines produced by immune cells and in turn produce pro-inflammatory cytokines amplifying the local immune responses through formation of a positive feedback loop, thereby contributing to sustain persistence of psoriasis lesions [3–5]. Hence, agents capable of inhibiting proliferation, inducing apoptosis of keratinocytes are potentially useful for psoriasis treatment [6–10].

Searching for novel agents for the treatment of psoriasis from natural products is always an important research direction for the development of anti-psoriatic strategy. Some natural compounds have been found to exhibit attractive anti-psoriatic potential based on the anti-proliferation activity [11–15]. Triterpenoids, widely distributed in the plant, have shown promising board-spectrum anti-proliferative activity [16]. Celastrol, a triterpene isolated from *Celastrus orbiculatus*, was found to induce the apoptosis of keratinocytes via downregulation of anti-apoptotic protein Bcl-2 and upregulation of the pro-apoptotic protein Bax [17]. Glycyrrhizin, a glycoconjugated triterpene exacted from licorice, displays an extensive range of biological activities [18]. Recently, glycyrrhizin was shown to exhibits anti-psoriatic efficacy through inhibiting NF-κB/ ICAM-1 signaling pathway in imiquimod (IMQ)-induced psoriasis-like mouse model [19]. Combined application of glycyrrhizin and anti-psoriatic drugs, including acitretin and methotrexate, improved clinical symptoms of psoriasis [20, 21]. 18β-Glycyrrhetinic acid (GA), a pentacyclic triterpene, is an active metabolite of glycyrrhizin, exhibiting a variety of biological activities similar to glycyrrhizin, including anti-inflammatory [22], anti-oxidant [23], anti-diabetic [24], anti-bacteria [25], cytoprotective effect [26, 27] and anti-proliferation effect [28, 29]. GA is thought to be primarily responsible for the pharmacological properties of glycyrrhizin [30]. However, the attempts to evaluate the anti-psoriatic activity of GA have not been reported. In this study, we tried to evaluate the effects of GA on the proliferation and apoptosis of human HaCaT keratinocytes in vitro and investigate the effects of GA on the skin lesions of imiquimod (IMQ)-induced psoriasis-like mouse model in vivo.

## Materials and methods

### Cell culture

HaCaT keratinocytes were obtained from Kunming Cell Bank of Type Culture Collection, Chinese Academy of Science (Kunming, China) and cultured in Dulbecco’s modified Eagle’s medium (DMEM) supplemented with 10% Fetal Bovine Serum (FBS) at 37 °C with 5% CO_2_.

### Chemicals and antibodies

18β-Glycyrrhetinic acid (GA) and LY294002 purchased from Sigma-Aldrich (St. Louis, Missouri, USA) were dissolved in DMSO. N-acetylcysteine (NAC) was purchased from Beyotime Institute of Biotechnology (Jiangsu, China) and dissolved in phosphate-buffered saline (PBS). Hydrogen peroxide (H_2_O_2_) was obtained from Sigma-Aldrich. Carbopol 940 and azone were purchased from Rhawn (Shanghai, China). IMQ cream was purchased from Sichuan Med-Shine Pharmaceutical Co., LTD (Sichuan, China).

Rabbit anti-Akt (pan), anti-p-Akt (Ser473) and anti-β-actin antibodies were purchased from Cell Signaling Technology (Beverly, MA, USA).

### Mice and treatment

C57BL6 mice (6–8 weeks old, female) obtained from Hunan SJA Laboratory Animal Co., Ltd. (Hunan, China) were maintained under a 12:12 light/dark cycle with free access to water and food. All experiments were conducted under ethics approval from Ethical Committees of Guilin Medical University (Guilin, China).

The composition of GA gel is as follows: GA 0.1 g, carbopol 940 0.2 g, azone 0.1 g, ethanol (96%) 3 g and distilled water q.s. to 10 g [31, 32]. The vehicle cream was also prepared without GA. Mice were randomly divided into four groups (*n* = 6 per group): Vaseline group (Ctl), IMQ group (IMQ), IMQ + vehicle group (IMQ + Vehicle) and IMQ + GA group (IMQ + GA). A daily topical dose of 62.5 mg of IMQ cream was used on the back skin of mouse for 14 consecutive days to induce psoriasis-like mouse model as described previously [33, 34]. A dose of 50 mg/cm^2^ GA cream or vehicle cream (without GA) was applied twice daily for 7 consecutive days at day 8–14 [32]. The severity of the psoriasis-like skin lesions was assessed on day 14 by using the Psoriasis Area Severity Index (PASI) [35]. Erythema, scaling and thickness were scored independently as follows: 0, none; 1, 2, moderate; 3, severe; 4, very severe. On day 14, the mice were euthanized and skin samples were collected for hematoxylin and eosin (H&E) staining and RNA extraction.

### RNA extraction and real-time PCR analysis

Total RNA was extracted from mice skin tissue using TRIzol™ reagent (Invitrogen, Carlsbad, USA) according to the manufacturer’s instruction. Reverse transcription was performed using RevertAid First Strand cDNA Synthesis Kit (Thermo Fisher Scientific, USA). CFX96 Touch™ Real-Time PCR Detection System (Bio-Rad, USA) was employed to perform real-time PCR (RT-PCR) with SYBR Green PCR Master Mix kit (TaKaRa, Japan). The procedure was as follows: 95 °C for 10 s, 60 °C for 10 s, 72 °C for 10 s, 40 cycles. GAPDH served as an internal reference. Primers sequences were as follows:

TNF-α, 5′-ATCCGCGACGTGGAACTG-3′ (forward) and.

5′-ACCGCCTGGAGTTCTGGAA-3′ (reverse);

IL-22, 5′-CAGCTCCTGTCACATCAGCGGT-3′ (forward) and.

5′-AGGTCCAGTTCCCCAATCGCCT-3′ (reverse);

IL-17A, 5′-CCTCACACGAGGCACAAGTG-3′ (forward) and.

5′-CTCTCCCTGGACTCATGTTTGC-3′ (reverse);

GAPDH, 5′-AGCTTGTCATCAACGGGAAG-3′ (forward) and.

5′-TTTGATGTTAGTGGGGTCTCG-3′ (reverse).

### Cell viability assay

Cell viability was measured using Cell Counting Kit-8 (CCK-8) (Dojindo, Japan). Briefly, HaCaT keratinocytes were seeded into 96-well plates. Cells with 60–80% confluence were treated with different doses of chemicals for 24 h. 10 μl of CCK-8 dye was added to each well of the plate and incubated for 2 h at 37 °C. Absorbance value at 450 nm was measured using Multiskan Spectrum (ThermoFisher) and directly proportional to the number of living cells. Besides, IC_50_ value was calculated by GraphPad Prism statistical software.

### Apoptosis analysis

HaCaT keratinocytes treated with different doses of chemicals for 24 h were harvested, washed with PBS and stained using propidium iodide (PI) and fluorescein isothiocyanate (FITC)-labeled annexin-V according to the manufacturer’s instructions (BD Pharmingen, USA). Apoptosis analysis was carried out using FACS Aria III flow cytometer (BD Biosciences).

### Caspase 9/3 activity measurement

The activities of caspase 9/3 were measured using caspase activity assay kits (Beyotime Institute of Biotechnology, China) according to the manufacturer’s instructions. Briefly, Cells were harvested and lysed. Total cellular protein concentration was measured with BCA kit (Pierce, USA). An equal amount of total protein extracts were incubated with Ac-LEHD-pNA (for caspase 9 assay) or Ac-DEVD-pNA (for caspase 3 assay) at 37 °C overnight. Absorbance value at 405 nm was measured using Multiskan Spectrum (ThermoFisher) and directly proportional to the activity of caspase 9/3.

### Measurement of ROS levels

HaCaT keratinocytes were treated with different doses of GA for 24 h. Cells were harvested, washed with PBS and incubated in 10 μM of 2′,7′-dichlorofluorescein diacetate (DCFH-DA, Sigma-Aldrich, USA) at 37 °C for 30 min. Subsequently, cells were washed with PBS for three times, and ROS levels were measured using FACS Aria III flow cytometer (BD Biosciences).

### Western blotting

HaCaT keratinocytes were treated with different doses of chemicals for 24 h. Cells were harvested and lysed. Total cellular protein concentration was measured with BCA kit (Pierce, USA). About 15 μg of protein was electrophoresed by SDS-PAGE and transferred onto PVDF membranes, which subsequently were blocked with 5% non-fat milk for 2 h at room temperature. After that, the membranes were incubated overnight at 4 °C with primary antibodies. Next, the membranes were washed with TBS-T for three times and incubated with HRP-conjugated secondary antibody for 2 h at room temperature. The protein signals were visualized using ChemiDoc™ XRS+ System (Bio-Rad, USA) with chemiluminescence substrate (Pierce, USA). The intensity of each band was measured using ChemiDoc™ XRS+ System software.

### Statistical analysis

All experiments were performed at least three times. GraphPad Prism statistical software was used for statistical analysis. Data were presented as mean ± standard deviation (SD). Statistical significance was analyzed using Student’s t test. *P* values < 0.05 was considered statistically significant.

## Results

### GA decreased cell viability in HaCaT keratinocytes

**Fig. 1:**
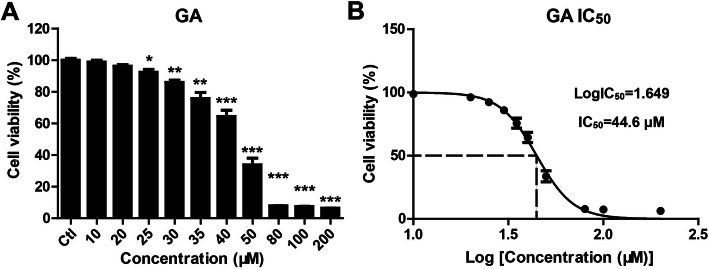
GA decreased cell viability in HaCaT keratinocytes. HaCaT keratinocytes were seeded in 96-well plates and treated with the indicated concentrations of GA for 24 h. (A) Cell viability was measured using CCK-8 assay. *P < 0.05, **P < 0.01 and ***P < 0.001 vs. control (Ctl) group. (B) IC_50_ value was calculated by GraphPad Prism statistical software.

To estimate the effect of GA on the cell viability of HaCaT keratinocytes, cells were seeded in 96-well plates and treated with the different concentrations of GA (0 for control, 10, 20, 25, 30, 35, 40, 50, 80, 100, 200 μM) for 24 h. Cell viability was measured using CCK-8 assay. GA at concentrations more than 25 μM treatment significantly decreased cell viability of HaCaT keratinocytes (Fig. 1a), with an IC_50_ value of 44.6 μM (Fig. 1b).

### GA induced apoptosis in HaCaT keratinocytes

**Fig. 2:**
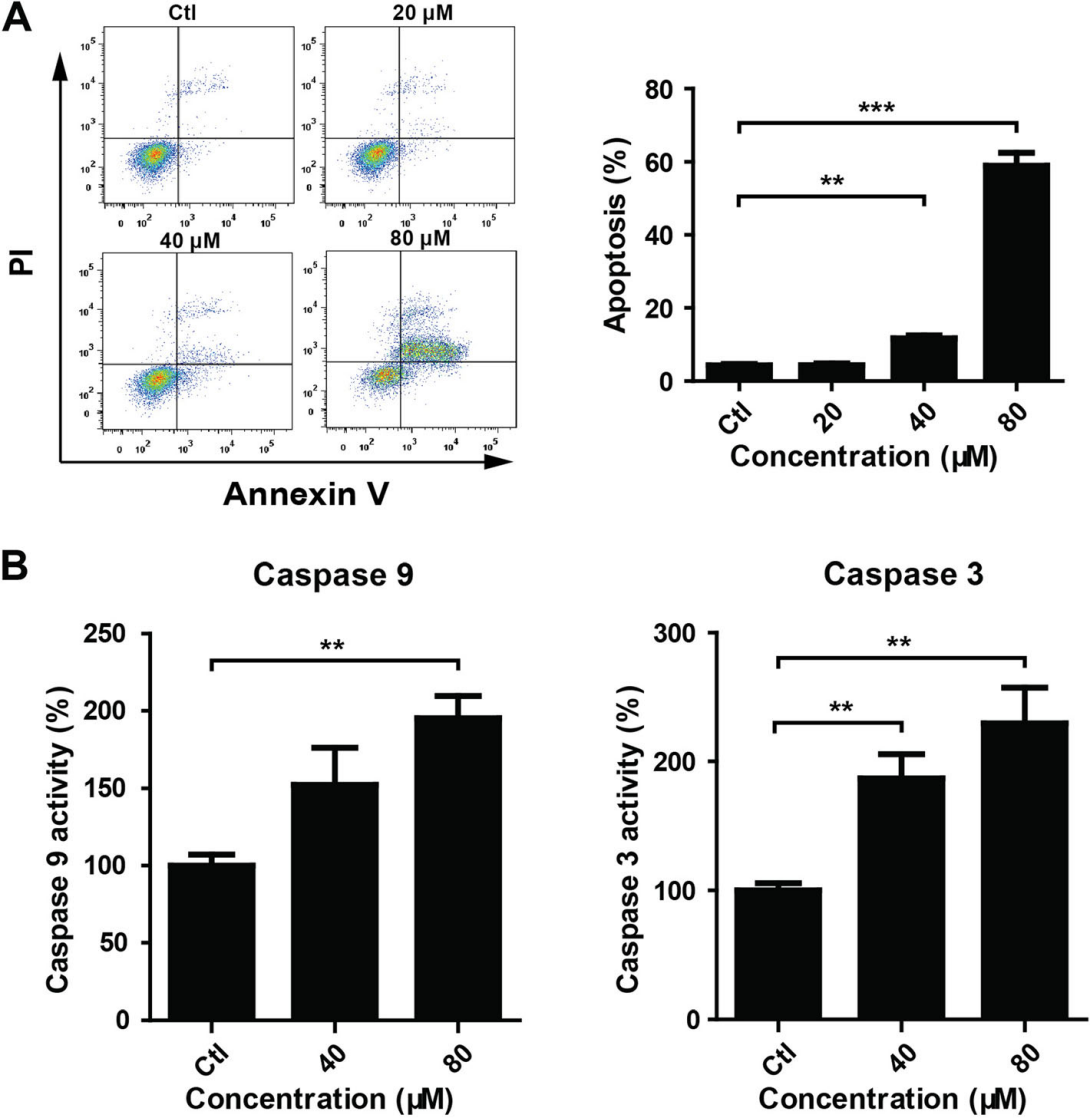
GA induced apoptosis in HaCaT keratinocytes. (A) HaCaT keratinocytes were seeded into 6-well plates and treated with GA (0 for control, 20, 40 and 80 μM) for 24 h. Cells were harvested and stained with PI and annexin V-FITC. Cell apoptosis was analyzed by flow cytometry. **P < 0.01. ***P < 0.001. (B) HaCaT keratinocytes were treated with GA (0 for control, 40 and 80 μM) for 24 h. Cells were harvested and lysed. Caspase 9 and 3 activities were determined using caspase activity assay kits. **P < 0.01

Flow cytometry was performed to evaluate the effect of GA on HaCaT keratinocytes apoptosis using PI and annexin V-FITC staining. GA (40 and 80 μM) treatment for 24 h dramatically increased the percentage of apoptosis cells (Fig. 2a). Consistently, GA (40 and 80 μM) treatment increased the activities of caspases 9 and 3 (Fig. 2b).

### GA induced ROS generation in HaCaT keratinocytes

**Fig. 3:**
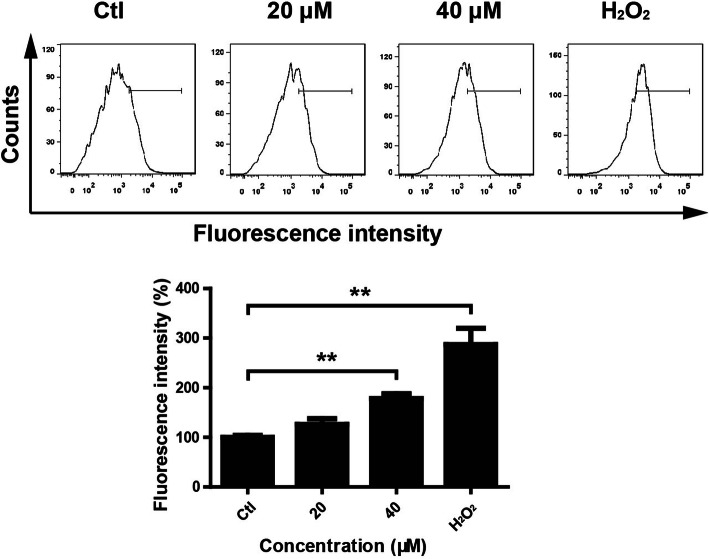
GA induced ROS generation in HaCaT keratinocytes. HaCaT keratinocytes were seeded into 6-well plates and treated with GA (0 for control, 20 and 40 μM) for 24 h. Cells were harvested and incubated with DCFH-DA. ROS levels were analyzed by flow cytometry. H_2_O_2_ treatment was used as a positive control. **P < 0.01

ROS plays important role in apoptosis induction under both physiologic and pathologic conditions [36]. In order to evaluate the effect of GA on ROS generation in HaCaT keratinocytes, DCFH-DA probe was used to detect ROS levels [37]. Flow cytometric assay showed that GA enhanced the fluorescence intensity (Fig. 3), indicating that GA treatment increased the accumulation of ROS in HaCaT keratinocytes.

### NAC treatment attenuated GA-mediated HaCaT keratinocytes growth inhibition and apoptosis

**Fig. 4:**
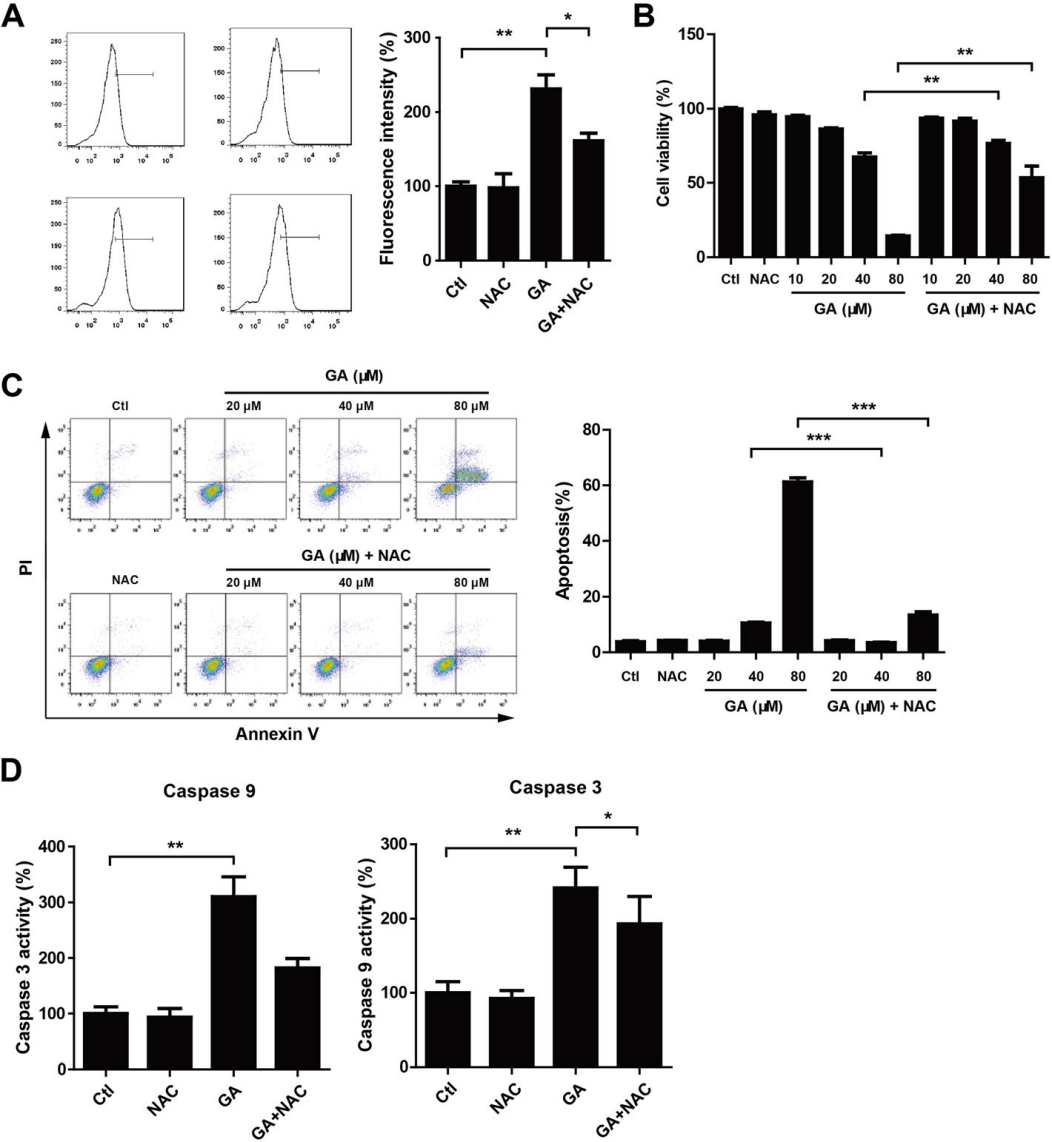
NAC treatment attenuated GA-mediated HaCaT keratinocytes growth inhibition and apoptosis. (A,D) HaCaT keratinocytes were seeded into 6-well plates and treated with GA (80 μM) and/or NAC (5 mM) for 24 h. Cells were harvested and incubated with DCFH-DA for detection of ROS levels by flow cytometry. Caspase 9 and 3 activities were determined using caspase activity assay kits. *P < 0.05. **P < 0.01. (B, C) HaCaT keratinocytes were treated with GA (indicated concentration) and/or NAC (5 mM) for 24 h. Cell viability was measured using CCK-8 assay. Cell apoptosis was analyzed by flow cytometry. **P < 0.01. ***P < 0.001

In order to explore the relationship between ROS-mediated apoptosis and ROS generation, NAC (a ROS inhibitor, 5 mM) was applied to inhibit ROS production. We observed that NAC could reduce GA-mediated increase of ROS generation (Fig. 4a) and partially restored GA-mediated decrease of HaCaT keratinocytes viability (Fig. 4b). Consistently, combined NAC and GA treatment reduced the percentage of apoptosis cells (Fig. 4c) and caspase 9/3 activities (Fig. 4d) when compared with GA treatment. These data suggested that GA-mediated apoptosis may be due to the accumulation of ROS in HaCaT keratinocytes.

### GA inhibited PI3K-Akt activation in HaCaT keratinocytes

**Fig. 5:**
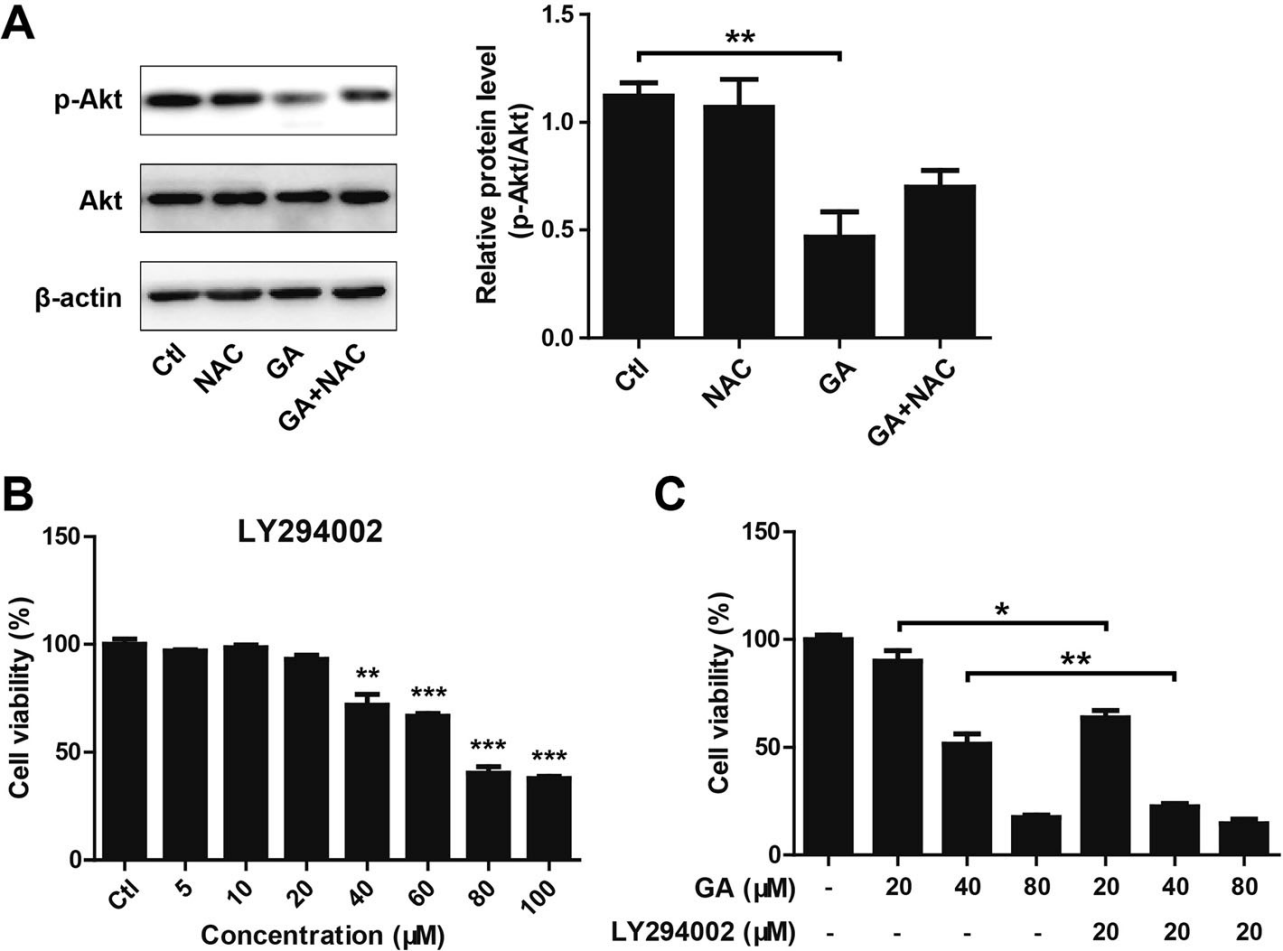
GA treatment inhibited PI3K-Akt activation in HaCaT keratinocytes. (A) HaCaT keratinocytes were seeded into 6-well plates and treated with GA (40 μM) and/or NAC (5 mM) for 24 h. Akt and p-Akt protein levels were detected by Western blotting. β-actin served as an internal reference. *P < 0.05. (B) HaCaT keratinocytes were seeded into 96-well plates and treated with different doses of LY294002 for 24 h. Cell viability was measured using CCK-8 assay. **P < 0.01 and ***P < 0.001 vs. control (Ctl) group. (C) HaCaT keratinocytes were seeded into 96-well plates and treated with GA (20, 40 and 80 μM) and/or LY294002 (20 μM) for 24 h. Cell viability was measured using CCK-8 assay. *P < 0.05. **P < 0.01

ROS-mediated apoptosis was shown to be closely associated with the inhibition of PI3K-Akt signaling pathway [38]. Therefore, Akt and p-Akt protein levels in HaCaT keratinocytes after treatment with GA, in presence of NAC or not, were detected by Western blotting. The results showed that GA treatment remarkably decreased p-Akt protein level (Fig. 5a). Interestingly, when the cells were co-treated with GA and NAC, p-Akt level was restored partially (Fig. 5a), indicating that GA-induced apoptosis in HaCaT keratinocytes may be due to ROS-mediated PI3K-Akt signaling inhibition. To further determine the involvement of PI3K-Akt signaling pathway into GA-induced apoptosis, LY294002 (a PI3K inhibitor) was used to inhibit PI3K-Akt signaling activation. No significant toxicity was observed in HaCaT keratinocytes treated with LY294002 for 24 h at concentration less than 20 μM (Fig. 5b). LY294002 (20 μM) treatment significantly enhanced the cytotoxicity of GA on HaCaT keratinocytes (Fig. 5c).

### GA ameliorated IMQ-induced psoriasis-like skin lesions in mice

**Fig. 6:**
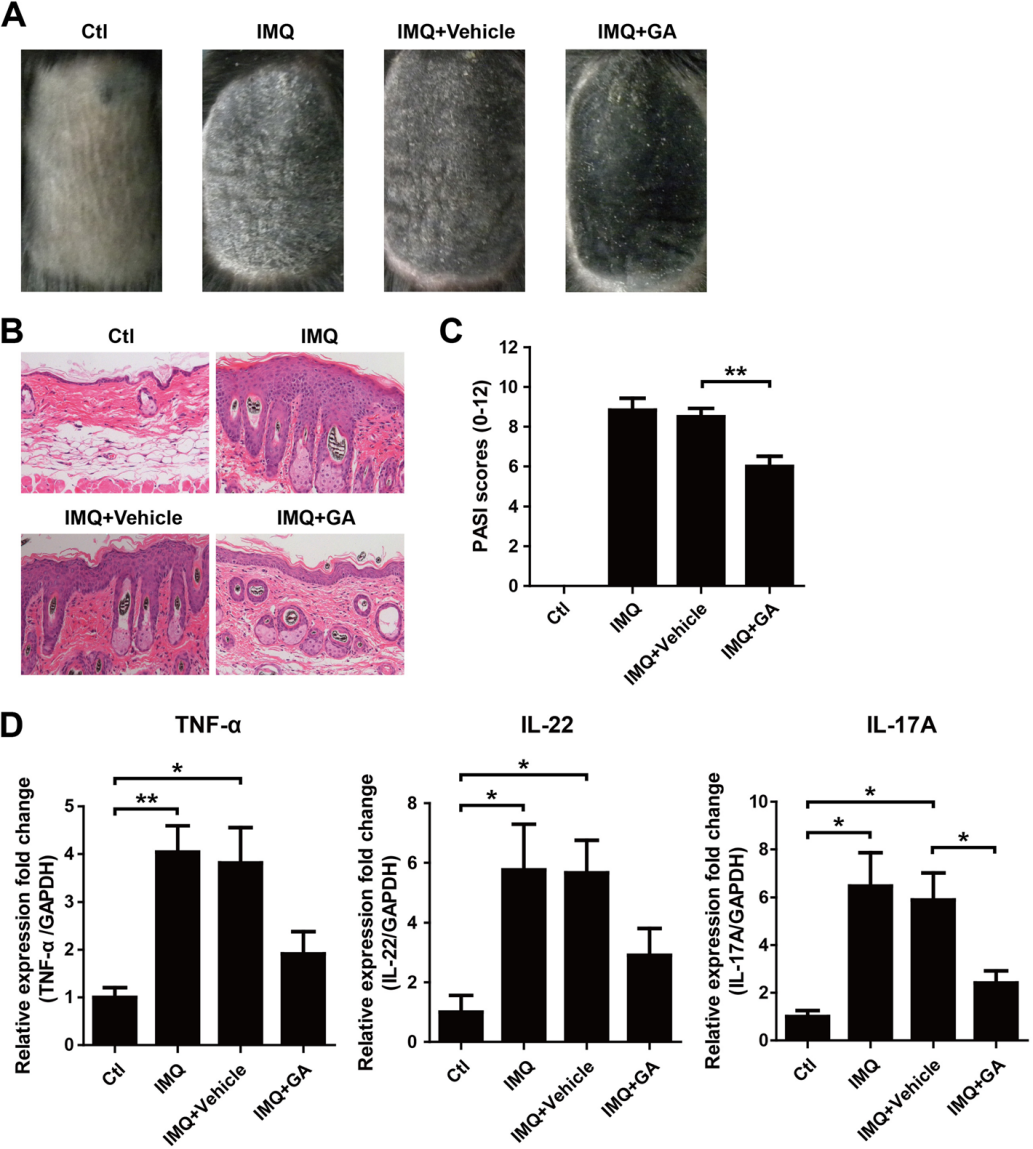
GA ameliorated IMQ-induced psoriasis-like skin lesions in mice. (A) The macroscopic appearance of mouse back skin on day 14. (B) H&E staining of the mouse skin on day 14. (C) PASI scores of mouse back skin lesions on day 14. **P < 0.01. (D) The relative expression of TNF-α, IL-22 and IL-17A in skin lesions on day 14 was measured using RT-PCR. GAPDH served as an internal reference. *P < 0.05. **P < 0.01

To evaluate the potential anti-psoriatic activity of GA in vivo, IMQ was applied topically on the skin of mouse to induce psoriasis-like lesions [33, 34]. Topical application of IMQ induced typical psoriatic characteristics in mice, including erythema, scaling, epidermis thickening and infiltration of immune cells, which could be improved in mice treated with GA cream, but no significant change was found in mice with vehicle cream (Fig. 6a-b). The severity of skin lesions (erythema, scaling and thickness) was scored based on PASI. Consistently, the mice treated with GA had lower score than that of vehicle group (Fig. 6c). In addition, the expression of inflammatory cytokines including TNF-α, IL-22 and IL-17A in skin lesions was measured using RT-PCR. The results showed that IMQ significantly up-regulated mRNA levels of TNF-α, IL-22 and IL-17A, which could be attenuated in mice treated with GA.

## Discussion

In skin, the balance between apoptosis and proliferation of keratinocytes maintains homeostasis of epidermis [39]. Diminished apoptosis and excessive proliferation of keratinocytes in psoriatic lesions have been found [6, 40], which is thought to be resulted from the abnormal crosstalk between keratinocytes and immune cells [3]. The hyperproliferative keratinocytes respond to a variety of cytokines in lesions, in turn, produce proinflammatory cytokines to exacerbate inflammatory response, creating a positive feedback loop [3–5]. Inhibiting keratinocytes proliferation is considered to be potentially useful for psoriasis management because the recovery from psoriatic to normal epidermis requires rebalanced homeostasis of keratinocyte growth and death [7, 10, 17, 41, 42]. The prescribed antipsoriatic drugs or strategies, such as methotrexate, dithranol, vitamin D3 derivatives and PUVA phototherapy, was shown to exert the therapeutic effect, to a great extent, through counteracting keratinocyte hyperproliferation or regulating keratinocyte differentiation [10, 43].

Glycyrrhizin, a natural triterpenoid saponin isolated from the root of the licorice (*Glycyrrhiza glabra*), has been used to treat ulcer, hepatitis and allergic skin diseases [30]. Recently, glycyrrhizin was found to ameliorate imiquimod-induced psoriasis-like skin lesions in mice [19], and improve clinical symptoms when combined with acitretin via reducing Th17 cell differentiation [20]. Given the fact that glycyrrhizin, with poor oral bioavailability, is primarily absorbed as glycyrrhetinic acid after hydrolysis of its carbohydrate moiety by intestine bacteria [44], therefore GA was thought to be primarily responsible for the pharmacological properties of glycyrrhizin [30, 44]. GA was found to significantly inhibit cell proliferation through inducing apoptosis in several cancer cells [45–47]. Interestingly, GA showed cytoprotective effect in PC12 and epithelial cells [26, 48], indicating different effects on viability in different cells. In the present study, we revealed that GA treatment significantly decreased HaCaT keratinocytes viability and induced apoptosis.

As known, ROS could induce apoptosis through activation of mitochondrial-mediated intrinsic apoptotic pathway [49]. In the current study, we found that GA induced ROS generation and increased the percentage of apoptosis cells in a dose-dependent manner. NAC, a ROS inhibitor, could attenuate GA-induced apoptosis of HaCaT keratinocytes, suggesting that GA induced apoptosis of HaCaT keratinocytes in a ROS-dependent manner. Traditionally, the increased ROS production and deficient function of antioxidant system are thought to be involved in the pathogenesis of many inflammatory skin diseases, including psoriasis [50–52]. However, the accumulating evidences suggested the protective role of ROS in immune-mediated diseases [53]. The deficiency of NADPH oxidase (NOX)-2 reduced ROS production and enhanced autoimmunity arthritis [54]. On the contrary, the deficiency of glutathione peroxidase-1 (GPx-1) increased the ROS level and attenuated allergen-induced airway inflammation [55]. Exacerbation of disease severity was observed in mice deficient for generation of ROS in mannan-induced psoriasis-like model, whereas, restoration of ROS production ameliorated both skin and joint disease [56]. Recently, ROS was shown to prevent IMQ-induced psoriatic dermatitis through enhancing regulatory T cell function [57]. Taken together, it was implied that GA-induced appropriately ROS in keratinocytes might be beneficial for psoriasis treatment.

ROS-induced apoptosis was proved to be largely associated with inhibition of PI3K-Akt signaling pathway [38]. Multiple cytotoxic agents exert pharmacological activities through ROS-mediated inhibition of PI3K-Akt signaling pathway [58–61].

PI3K-Akt signaling, a crucial pathway responsible for regulating survival signals [62], was reported to be hyper-activated in the keratinocytes in psoriasis lesions [63, 64], which subsequently phosphorylated the downstream target proteins FOXO and mTOR, thereby promoting keratinocytes proliferation [65–68]. Reasonably, it is thought that the inhibition of PI3K/Akt signaling pathway could be promising anti-psoriatic strategy [69–71]. In the current study, we found that GA treatment decreased p-Akt protein level. Whereas, combined with NAC treatment, p-Akt level was restored partially. In addition, LY294002, a PI3K inhibitor, significantly enhanced GA-mediated cytotoxicity. These results indicated that GA-induced apoptosis in HaCaT keratinocytes may be due to the inhibition of ROS-mediated PI3K-Akt signaling.

IMQ, an agonist of Toll-like receptor 7/8 ligand, is widely topically applied on the skin of mouse to induce psoriasis-like lesions closely resembles human psoriasis in phenotypic and histological characteristics [33]. In order to better function on kerationcytes, GA was applied topically on the skin of mouse. In the current study, we found that GA ameliorated IMQ-induced psoriasis-like skin lesions, including phenotypic and histological characteristics. TNF-α, an important proinflammatory cytokine, was found to exert great function in psoriasis pathogenesis [72, 73]. IL-17 and IL-22, two Th17-related cytokines, was shown to be involved in maintaining chronic inflammatory response and remodeling epithelial tissues [74–76]. In this study, we observed that GA treatment attenuated IMQ-induced upregulation of TNF-α, IL-22 and IL-17A in mouse skin lesions. Taken together, these findings highlight the potential application of GA for the treatment of psoriasis. However, further studies will be required to identify the targets of GA, and figure out how these interactions might regulate keratinocytes proliferation.

## Conclusions

In conclusion, GA can decrease HaCaT keratinocytes viability and induce cell apoptosis in a dose-dependent manner. GA-mediated HaCaT keratinocytes apoptosis is associated with ROS induction and consequent inhibition of PI3K-Akt signaling pathway. In addition, GA ameliorates IMQ-induced psoriasis-like skin lesions in mice. These results provide experimental evidences to support further developing GA as an anti-psoriatic agent.

## Data Availability

All data generated or analyzed during this study are included in this published article.

## Abbreviations

**GA**: 18β-Glycyrrhetinic acid
**NAC**: N-acetylcysteine
**DCFH-DA**: 2′,7′-dichlorofluorescein diacetate
**DMSO**: Dimethyl sulfoxide
**H**_**2**_**O**_**2**_: Hydrogen peroxide
**IMQ**: Imiquimod
**PASI**: Psoriasis Area Severity Index
**FITC**: Fluorescein isothiocyanate
**PI**: Propidium iodide
**SDS-PAGE**: Sodium dodecyl sulfate-polyacrylamide gel electrophoresis
**PVDF**: Polyvinyl difluoride
**CCK-8**: Cell Counting Kit-8
**PBS**: Phosphate-buffered saline
**TBS-T**: Tris-buffered saline containing 0.5% Tween-20
**PMSF**: Phenylmethyl-sulfonylfluoride
**PI3K**: Phosphatidylinositol 3 kinase
**Akt**: Serine-threonine kinase
**ROS**: Reactive oxygen species
